# Immunohistochemical detection of S100A1 in the postmortem diagnosis of acute myocardial infarction

**DOI:** 10.1186/1746-1596-8-84

**Published:** 2013-05-17

**Authors:** Haitao Bi, Ying Yang, Jianye Huang, Yingmin Li, Chunling Ma, Bin Cong

**Affiliations:** 1 Department of Forensic Medicine, Hebei Medical University, No. 361 Zhongshan Road, Shijiazhuang, Hebei 050017, China

**Keywords:** Acute myocardial infarction, S100A1, Immunohistochemistry, Enzyme-linked immunosorbent assay

## Abstract

**Background:**

Sudden cardiac death resulting from acute myocardial infarction (AMI) constitutes a significant percentage of the caseload for forensic and clinical pathologists. When sudden death occurs at an early stage (<6 h), pathologists experience difficulty in the postmortem diagnosis of AMI. Because of the specific tissue distribution of S100A1 and its relationship with acute ischemic heart disease, this study aimed to evaluate the performance of S100A1 in the postmortem diagnosis of AMI.

**Methods:**

We constructed a rat model of AMI through permanent ligation of the left anterior descending coronary artery (LAD) to investigate the depletion of S100A1 from ischemic cardiomyocytes by immunohistochemistry and measuring S100A1 plasma concentrations by enzyme-linked immunosorbent assay at varying post-infarction intervals. In addition, immunohistochemical staining of S100A1 for definite infarction, suspected early infarction, and in normal human hearts, was also performed to test its practical feasibility for postmortem diagnosis of AMI at an early stage.

**Results:**

As early as 15 min after ligation of the LAD, depletion of S100A1 was observed in ischemic cardiomyocytes, and S100A1 plasma concentration was also significantly higher than that of the sham-operated group (*P* < 0.001). With continuation of the occlusion time, the depleted areas of S100A1 further expanded and S100A1 plasma concentrations further increased. For autopsy material, all human cases of definite myocardial infarction and suspected early infarction showed well-defined areas without S100A1 staining. None of the normal human cases showed diffuse depletion of S100A1.

**Conclusion:**

Our results suggest that immunohistochemical detection of S100A1 is useful for the postmortem diagnosis of AMI at an early stage.

**Virtual slides:**

The virtual slide(s) for this article can be found here:

http://www.diagnosticpathology.diagnomx.eu/vs/4366650979519818

## Background

Sudden cardiac death (SCD) is most commonly defined as unexpected death from a cardiac cause within a limited time period, generally <1 h from symptom onset [1]. SCD resulting from acute myocardial infarction (AMI) constitutes a significant percentage of the caseload for forensic and clinical pathologists [2]. AMI is defined in pathology as myocardial cell death due to prolonged ischemia [3]. Patients presenting with symptoms of AMI are often subject to a standardized battery of tests, including a radiological examination, an electrocardiogram, and blood samples submitted for cardiac markers, such as cardiac troponin (cTn) and creatine kinase MB (CK-MB). Unfortunately, the postmortem diagnosis of AMI for forensic and clinical pathologists is difficult. Myocardial cell death does not occur instantaneously at the onset of ischemia, but takes at least 6 h before myocardial necrosis can be identified by standard macroscopic or microscopic postmortem examination, depending on the sensitivity of the cardiomyocytes [4]. Although many markers and various techniques have been introduced for postmortem diagnosis of AMI, currently, there is neither a marker nor a technique in routine medicolegal use that can solve this problem satisfactorily [4-6].

S100A1, a dimeric Ca^2+^-binding protein of the EF-hand type, belongs to the S100 protein family [7]. As a low molecular weight protein (~10.5 kDa) with a specific tissue distribution [8], S100A1 is preferentially abundant in the heart, especially ventricular cardiomyocytes [9], although it is also found in lower amounts in skeletal muscle, blood vessels, brain, and kidney [10-12]. A compelling body of evidence has disclosed a role for S100A1 as a critical regulator of cardiomyocyte Ca^2+^ cycling, energy homeostasis, and excitation-contraction coupling. S100A1 is especially interesting with respect to cardiovascular diseases because downregulation of S100A1 protein critically contributes to the progressive contractile dysfunction of the diseased heart and cardiac-related death [13,14]. Shortly after ischemic myocardial damage in humans, S100A1 appears in the serum, rising rapidly after the clinical onset [15,16].

Because of S100A1’s specific and differential expression in the heart and relationship to acute ischemic heart disease, we tested its performance as a diagnostic indicator of AMI. In the present study, we constructed a rat myocardial infarction model to investigate the temporal and spatial distribution of S100A1 in cardiomyocytes and S100A1 plasma concentrations after AMI. Immunohistochemical staining for S100A1 in definite infarction, suspected early infarction, and normal human hearts was also performed to test its practical feasibility.

## Materials and methods

### Experimental animals

Healthy Sprague–Dawley rats (250–300 g) of either sex provided by the Laboratory Animal Department of Hebei Medical University were used for the study. The rats were bred and cared for under standard laboratory conditions, and had *ad libitum* access to food and water. All animal procedures were approved by the Institutional Animal Care and Use Committee. The following groups of rats were studied to evaluate depletion of S100A1 in cardiomyocytes and the leakage of cytoplasmic S100A1 into the blood circulation after AMI: (1) sham-operated group (n = 10); (2) 15 min after left anterior descending coronary artery (LAD) occlusion (n = 10); (3) 30 min after LAD occlusion (n = 10); (4) 1 h after LAD occlusion (n = 10); (5) 2 h after LAD occlusion (n = 10); (6) 4 h after LAD occlusion (n = 10); and (7) 6 h after LAD occlusion (n = 10).

### Experimental induction of myocardial infarction

The animal model of AMI was induced surgically by permanent ligation of the LAD as previously described [17]. Animals were anesthetized with 350 mg/kg chloral hydrate, intubated, and ventilated with an animal ventilator (Model ALC-V8, China). Following thoracotomy, the pericardium was carefully opened, avoiding any injury to the heart, and the LAD was ligated with a 6/0 silk suture by piercing the pericardial membrane. Successful ligation was tested by visual inspection for pallor of the involved myocardium and ST segment elevation ≥0.1 mv on an electrocardiogram. The thorax was closed in layers, and the lungs were reinflated using positive end-expiratory pressure. The endotracheal tube was removed and the animals were returned to normal respiration. The sham-operated animals were treated similar to the study groups, except that they did not receive LAD ligation. After a blood sample was collected, rats ware humanely sacrificed under general anesthesia at various ischemia intervals. The hearts were harvested and fixed in a 4% phosphate-buffered (pH 7.4) formaldehyde solution over 24 h. The samples were sequentially dehydrated with an alcohol series and embedded in paraffin wax. Five-micrometer sections were prepared from paraffin blocks and stained with hematoxylin and eosin (H&E).

### Autopsy material

Paraffin-embedded myocardial tissue blocks were obtained from 30 autopsies performed between 2009 and 2013 at the Department of Forensic Medicine of Hebei Medical University. The age at autopsy ranged from 22 to 78 years (mean, 51 years) and the postmortem intervals varied from 8 to 72 h. Myocardial tissues for examination were taken from the anterior part of the left ventricle, including the zone of definite or possible infarction. The cases were divided into three groups as follows. (1) Group 1 included 10 cases of definite AMI, which was proven at gross examination (coronary occlusion) and at conventional histology with H&E (necrotic clotting, multifocal patches of wavy fibers, contraction band necrosis, and marginal rearrangement reactions with early inflammatory infiltrate). (2) Group 2 included 10 cases of acute traumatic death, which were selected among victims of traffic accidents with immediate lethal craniocerebral injuries or falls from height with no or minimal signs of coronary atherosclerosis. (3) Group 3 included 10 cases of suspected early myocardial infarction, and met the following criteria: (i) death occurred within 6 h from the onset of symptoms, which indicated AMI clinically; (ii) the subjects had a moderate or severe degree of coronary atherosclerosis at autopsy, but none of them had macroscopic or microscopic (H&E staining) evidence of myocardial infarction; and (iii) no other explainable causes of death were found through a systemic examination, including systemic autopsy and toxicological analysis.

### Hematoxylin-basic fuchsin-picric acid (HBFP) staining

To confirm early myocardial ischemic damage, 5-μm sections were prepared and stained by HBFP staining [18], which stained ischemic cardiomyocytes crimson red and normal cardiomyocytes yellow.

### Immunohistochemical staining for S100A1

Immunohistochemical staining of 5-μm sections was performed using the Two-step IHC Detection Reagent (ZSGB-BIO, China). Briefly, after being treated with microwave antigen retrieval (0.1 M citrate buffer solution, pH 6.0) for 5 min, sections were pre-treated with 0.3% H_2_O_2_ in methanol for 30 min to inhibit endogenous peroxidase activity. Blocking solution with 10% goat serum (Boster, China) was then applied to the sections for 30 min at room temperature to minimize non-specific staining. Subsequently, they were incubated overnight at 4°C with polyclonal rabbit anti-S100A1 (BS1318, Bioworld, USA) at a dilution of 1:600. PBS was used to replace S100A1 antibody as the negative staining control. Labeling was identified by application of a goat anti-rabbit IgG/horseradish peroxidase secondary antibody (PV-6001, ZSGB-BIO, China) at 37°C for 30 min. Peroxidase activity was visualized using a DAB kit (ZSGB-BIO, China). The reaction was stopped by rinsing in PBS. Sections were weakly counterstained with hematoxylin. Finally, the slides were dehydrated, placed in an aqueous-based mounting medium, and examined by light microscopy.

### Immunoassay for S100A1

Blood samples were collected via retro-orbital sinus puncture with a capillary tube before rats were sacrificed. The blood samples were centrifuged at 3000 rpm for 20 min at 4°C, and the serum was then separated and stored at -80°C until later assay. Serum concentrations of S100A1 were measured using an ELISA kit (Hebei Bio-high Technology, China) according to the manufacturer’s protocol. Briefly, 50 μl of the biotinylated antibody against rat S100A1 and 50 μl of either the diluted plasma or the S100A1 standards were added to the precoated plate. After incubation for 2 h at room temperature, the wells were washed five times with wash buffer followed by the addition of 50 μl streptavidin-HRP. After incubation for an additional 2 h at room temperature, the washing steps were then repeated as described above, followed by adding 100 μl of substrate solution to each well. The microplate was allowed to stand for 30 min at room temperature in the dark. The reaction was stopped by adding 50 μl stop solution and the absorbance at 450 nm was measured using an ELx800 Absorbance Microplate Reader (BIO-TEK Instruments, Inc., USA). S100A1 concentrations for each sample were calculated from a standard curve.

### Statistical analysis

Data are described as the median (interquartile range). Statistical analyses were performed by the Kruskal-Wallis *H* test, followed by comparison between any two groups using SPSS 13.0 software. *P* values lower than 0.05 were considered statistically significant.

## Results

### H&E staining of cardiomyocytes among the groups

**Figure 1:**
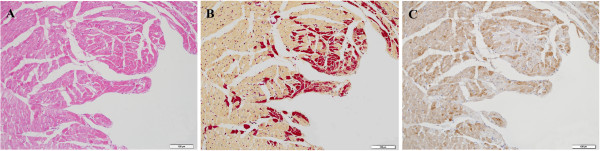
**H&E and HBFP staining, and immunohistochemical staining for S100A1 15 min after ligation. A**: The H&E stained sections showed conservation of normal morphology of cardiomyocytes, except for a few cardiomyocytes in the subendocardial layer and papillary muscles, with a weakly eosinophilic cytoplasm (bar: 100 μm). **B**: Ischemic cardiomyocytes in the subendocardial layer and papillary muscles were positively stained crimson in contrast to yellow for nonischemic cardiomyocytes (HBFP, bar: 100 μm). **C**: Depletion of S100A1 in a few cardiomyocytes was detected by IHC, which was in agreement with the positively stained areas of HBFP (bar: 100 μm).

**Figure 2:**
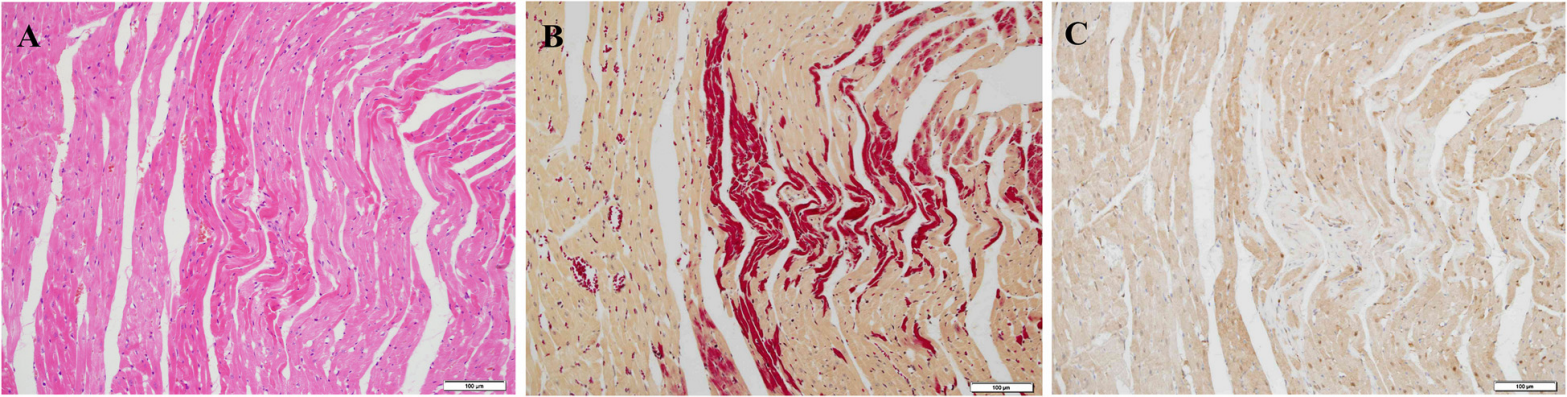
**H&E and HBFP staining, and immunohistochemical staining for S100A1 2 h after ligation. A**: Partial wavy cardiomyocytes with eosinophilic cytoplasm were observed in ischemic areas (bar: 100 μm). **B**: The positively stained areas of HBFP extended to the middle myocardium (bar: 100 μm). **C**: The positively stained areas of HBFP showed significant depletion of S100A1 (bar: 100 μm).

The H&E-stained sections of 15 min post AMI showed conservation of normal morphology of cardiomyocytes, except for a few cardiomyocytes in the subendocardial layer and papillary muscles, with a weakly eosinophilic cytoplasm (Figure 1A). By 2 h after ligation, partial wavy cardiomyocytes with an eosinophilic cytoplasm were observed in ischemic areas (Figure 2A). By 6 h after ligation, the H&E-stained sections showed local interstitial edema and multifocal patches of wavy fibers with increased eosinophilia of the cytoplasm in ischemic areas.

### HBFP staining of cardiomyocytes among the groups for confirmation of early myocardial damage

Fifteen minutes after ligation, ischemic cardiomyocytes in the subendocardial layer and papillary muscles were positively stained crimson in contrast to yellow observed in nonischemic tissue (Figure 1B). When the ischemic time was prolonged, the positively stained areas of the heart expanded and extended to the middle of the myocardium 2 h after ligation (Figure 2B). By 4 h after ligation, crimson staining was observed in all infarcted myocardial tissue, which was distinct from normal cardiomyocytes.

### Immunohistochemical staining for S100A1 in rat hearts

In the sham-operated group, the cytoplasm of all cardiomyocytes was stained brown (positive for S100A1) and the nuclei were stained light blue. The intensity of staining was uniformly strong and diffusely distributed in all cardiomyocytes.

**Figure 3:**
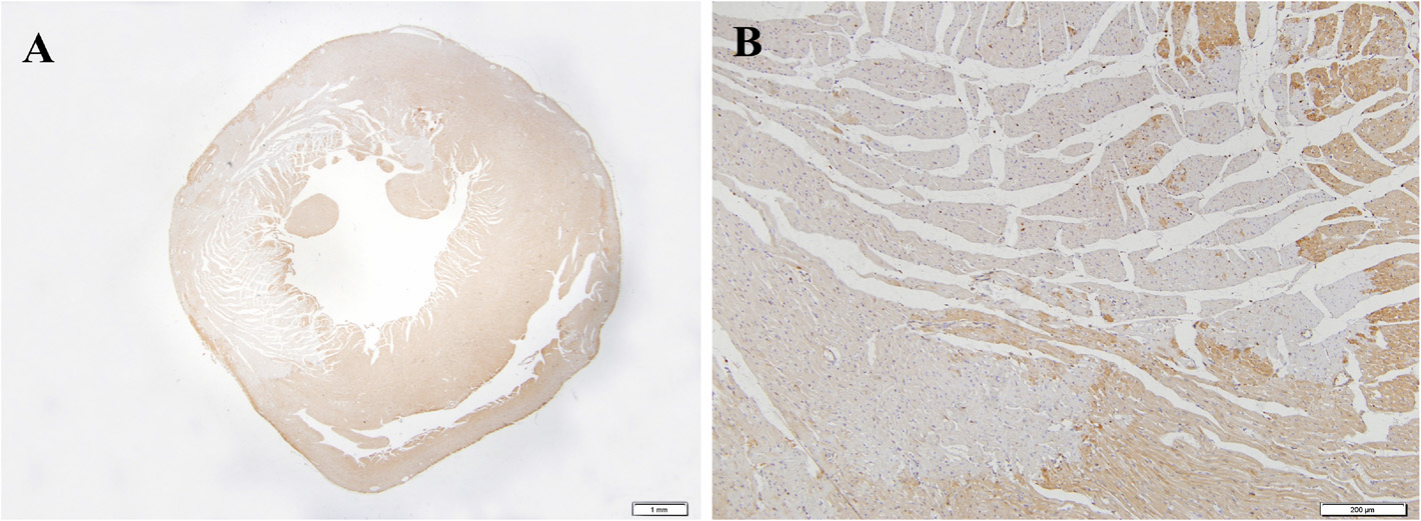
**Immunohistochemical staining for S100A1 4 h after ligation. **Complete depletion of S100A1 in the left ventricle within the LAD supply region was observed 4 h after LAD occlusion, and this was distinct from normal cardiomyocytes. (**A**: bar: 1 mm; **B**: bar: 200 μm).

In the study groups (AMI model), depletion of S100A1 occurred in a few cardiomyocytes in the subendocardial layer and papillary muscles 15 min after LAD occlusion (Figure 1C), which was consistent with the positively stained areas of HBFP. Thirty minutes after LAD occlusion, cardiomyocytes with absent S100A1 staining still located in the subendocardial layer and papillary muscles. Areas of patchy depletion of S100A1 were larger at 1 h than at 30 min post AMI, and they extended to the middle myocardium by 2 h post AMI (Figure 2C). By 4 h after ligation, immunohistochemical staining showed well-demarcated areas of complete depletion of cytoplasmic staining of S100A1 in the left ventricle within the LAD supply region (Figure 3A, B). Six hours after LAD occlusion, the depleted areas of S100A1 had further expanded with the prolongation of infarction time.

### Plasma S100A1 concentrations after AMI

**Figure 4:**
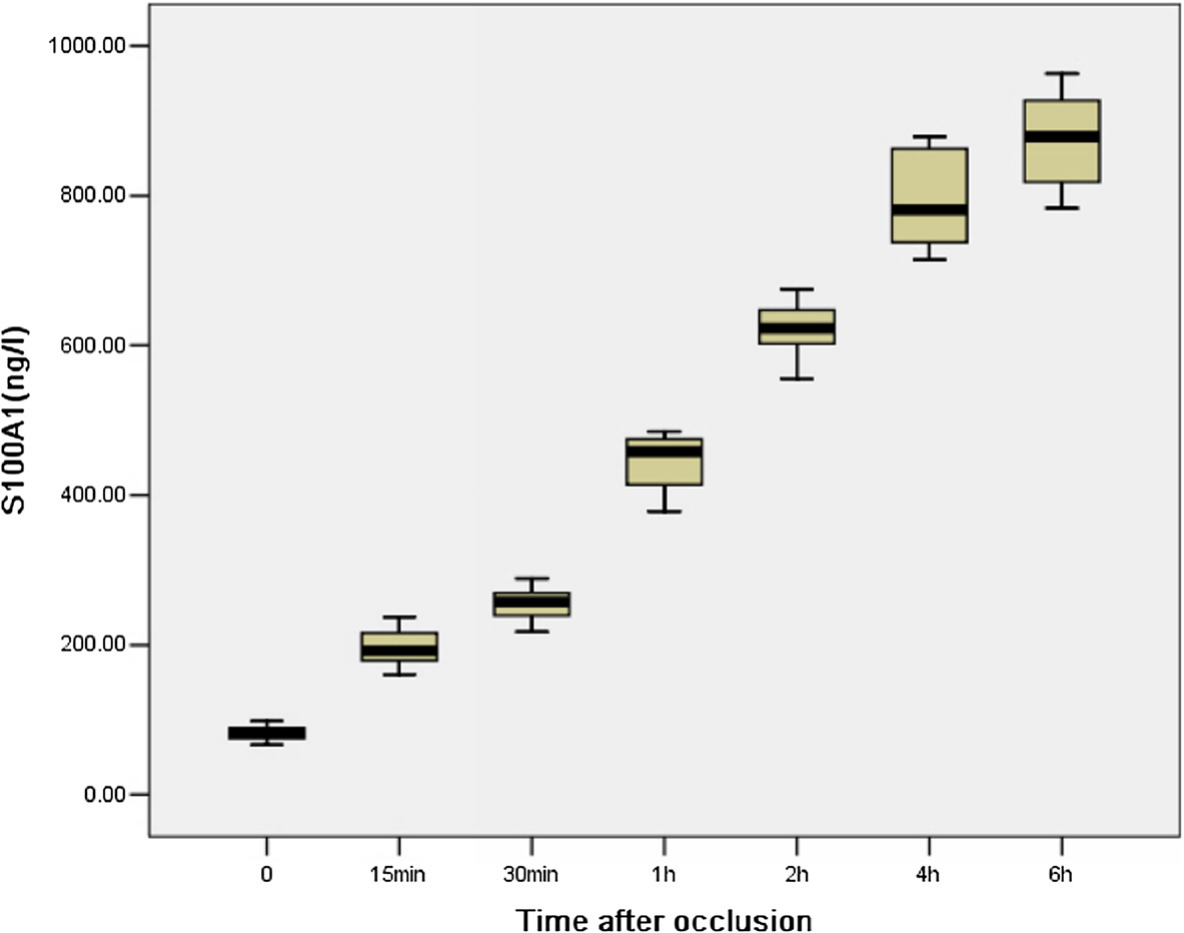
**Boxplot of plasma S100A1 concentrations in rats. **The median plasma S100A1 concentration in the sham-operated group was 81.98 ng/l (interquartile range: 15.7 ng/l). At 15 min after AMI, the median S100A1 concentration (192.86 [39.9] ng/l) was significantly higher than that of the sham-operated group (*P* < 0.001). With the continuation of occlusion time, plasma S100A1 concentrations further increased.

The time course of plasma concentrations of S100A1 after AMI is shown in Figure 4. The median plasma S100A1 concentration in the sham-operated group was 81.98 ng/l (interquartile range: 15.7 ng/l). At 15 min after AMI, the median S100A1 concentration (192.86 [39.9] ng/l) was significantly higher than that of the sham-operated group (*P* < 0.001). With the continuation of occlusion time, plasma S100A1 concentrations further increased. Plasma S100A1 concentrations were 257.27 ng/l (33.19 ng/l), 458.34 ng/l (62.73 ng/l), 622.84 ng/l (53.17 ng/l), and 781.70 ng/l (133.35 ng/l) 30 min, 1 h, 2 h, and 4 h after LAD occlusion, respectively, and peaked at 6 h (878.57 ng/l [119.38 ng/l]).

### Immunohistochemical staining for S100A1 in human hearts

**Figure 5:**
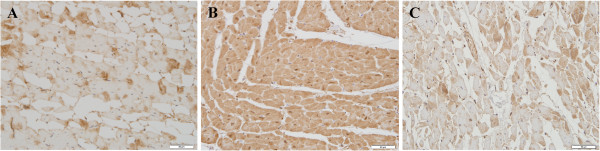
**Immunohistochemical staining for S100A1 in human hearts. A**: Massive depletion of H-FABP in ischemic areas was observed (bar: 50 μm). **B**: Strong cytoplasmic S100A1 immunoreactivity can be seen in normal cardiomyocytes (bar: 50 μm). **C**: A significant reduction in S100A1 expression in ischemic areas was observed (case 5, bar: 50 μm).

In all 10 cases of group 1 with definite infarcted areas, there was significant depletion of S100A1 in fibrous tissue and massive depletion in ischemic areas (Figure 5A). In group 2, strong cytoplasmic S100A1 immunoreactivity was observed in cardiomyocytes (Figure 5B), although some staining was light. In group 3, four cases showed massive depletion of S100A1 expression in ischemic areas (Figure 5C). Four cases showed patchy depletion of S100A1 and two cases showed sparse loss in a few cardiomyocytes. S100A1 immunostaining results in group 3 are shown in Table 1.

**Table 1:** Patient characteristics and S100A1 immunostaining results in 10 human heart samples of group 3

**Case**	**Sex**	**Age (years)**	**Duration of symptoms**	**Postmortem interval (h)**	**Coronary atherosclerosis (% of narrowing)**^**☆**^	**loss of S100A1**^**★**^
1	M	59	1 h	36	IV	++
2	M	22	?	19	IV	+
3	M	55	30 min	24	IV	±
4	M	62	45 min	24	III	+
5	M	68	2 h	30	IV	++
6	M	58	2.5 h	10	III	++
7	F	64	1 h	48	IV	+
8	M	35	1 h	72	IV	+
9	M	44	3 h	42	III	++
10	M	48	30 min	24	IV	±

^☆^Grade of coronary atherosclerosis: Grade I: 0–25% of narrowing; Grade II: 25–50%; Grade III: 50–75%; Grade IV: >75%.

^★^Loss of staining is scaled as: massive loss (++); patchy loss (+); loss in a few disseminated cells (±).

## Discussion

The most common clinical finding associated with SCD is coronary artery disease (CAD) and approximately 80% of SCDs are attributed to this disease condition [19]. Another 10% to 15% of SCDs result from cardiomyopathies, such as hypertrophic cardiomyopathy, arrhythmogenic right ventricular cardiomyopathy [20], and myocardial infiltrative diseases. The remaining 5% to 10% are composed of structurally abnormal congenital cardiac conditions (i.e., coronary artery abnormalities [21,22]), cardiac channelopathies and relatively rare diseases such as cardiac beriberi caused by Thiamine deficiency [23]. CAD associated with occlusion of one or more major coronary arteries is likely to result in ventricular tachyarrhythmia. This arrhythmia, if untreated, will eventually degenerate into ventricular fibrillation, which is the underlying mechanism in the overwhelming majority of SCDs [19,24]. AMI is generally the result of CAD. When sudden death occurs at an early stage of AMI (< 6 h from the onset of symptoms to death), myocardial cell death cannot easily be detected by routine histologic techniques, such as H&E staining. These individuals die before pathological changes can develop in the myocardium, and it is difficult for the clinical and forensic pathologists to say with certainty whether these patients succumbed to AMI.

Irreversible cardiomyocyte injury in AMI can be recognized by the appearance of cardiac proteins in the bloodstream, which are released into the circulation from injured cardiomyocytes, because of increased permeability of the myocardial cell membrane associated with severe ischemia [25]. The temporal release pattern of these cellular proteins is dependent on the extent of hypoxia, their subcellular localization, and their physicochemical characteristics, especially molecular weight. Therefore, cellular antigens, such as MB [4], cTnI [26], and H-FABP [17], can indicate early myocardial infarction based on the loss of staining in infarcted areas.

Given the low molecular weight and specific tissue distribution of S100A1, S100A1 may leak from damaged cardiomyocytes into the bloodstream of patients with AMI. Therefore, S100A1 could be used as a “negative marker” to discern ischemic-damaged myocardial cells from normal cells, which could be helpful for postmortem diagnosis of AMI. Previous studies [15,16] have shown that serum S100A1 concentrations are significantly elevated after AMI and have higher sensitivity and specificity than MB and cTnI within the early hours (0–6 h) of the onset of myocardial infarction. The prompt release of S100A1 into the bloodstream most likely reflects irreversible changes in cardiomyocytes due to hypoxia. However, little is known regarding the expression pattern of S100A1 in ischemic cardiomyocyte lesions.

In the present study, immunohistochemical results of the AMI animal model showed that as early as 15 min after myocardial ischemia, S100A1 depletion was detected in the subendocardial layer and papillary muscles, which was in agreement with the positively stained areas of HBFP. When the ischemic time was prolonged, this depletion was increasingly evident. By 4 h after ligation, the staining showed well- demarcated areas of complete depletion of cytoplasmic staining of S100A1 in the left ventricle within the LAD supply region. Our results showed a time-dependent depletion of S100A1 after AMI. Additionally, depletion of S100A1 from cardiomyocytes in infarcted areas at various post-infarction intervals showed an early identical pattern with that of H-FABP [17]. These results strongly suggest that depletion of S100A1 staining in cardiomyocytes can be used as a sensitive and reliable marker for AMI.

ELISA results showed a prolonged increase in serum S100A1 concentrations, providing evidence that S100A1 is released from injured cardiomyocytes after AMI. Kiewitz et al. [16] demonstrated that the concentration-time course of S100A1 is distinct from that of the “classical” biochemical markers CK, CKMB, and cTnI, showing an early rise and a fast decline in plasma after the ischemic event. The sensitivity of S100A1 between 0 and 6 h is significantly higher than that from 6 to 12 h compared with cTnI. The present study showed that S100A1 plasma concentrations were low in the sham-operated group, but they were significantly higher as early as 15 min after occlusion of the rat LAD than those in the sham-operated group, indicating its high diagnostic sensitivity for AMI. Moreover, S100A1 plasma concentrations increased with ischemia, and reached a peak after 6 h, which was consistent with the results of IHC. This finding suggests that plasma S100A1 concentrations are in direct proportion to the extent of ischemic cardiomyocyte injury.

In the autopsy material, the efficacy of S100A1 for detection of ischemic cardiomyocyte lesions appeared to be satisfactory. All cases of definite AMI showed well-defined areas with a significant reduction or total loss of S100A1 expression. In group 2, eight of 10 cases with the conclusion of “possible AMI” showed massive or patchy depletion of S100A1. In only two cases (cases 3 and 10), because of the short duration from the onset of symptoms to death (30 min), S100A1 expression was only lost in a few disseminated cells. These S100A1 immunostaining results indicated that depletion of S100A1 could be detected as early as 30 min after ischemic lesions in the human heart. This finding strongly suggests that depletion of S100A1 from cardiomyocytes is a useful marker for the postmortem diagnosis of AMI for forensic and clinical pathologists.

No single immunohistochemical reaction is ideal for the postmortem diagnosis of AMI, and a reasonable combination of several markers can provide sufficient evidence of myocardial necrosis, supporting the final diagnosis. Our study results indicate that evaluation of immunohistochemical expression of S100A1 can complement the presently used markers to improve the postmortem diagnosis of AMI.

## Conclusions

In conclusion, depletion of S100A1 was observed in ischemic cardiomyocytes. The results of this study indicate that immunohistochemical detection of S100A1 is useful for the postmortem diagnosis of AMI at an early stage.

## Abbreviations

AMI: Acute myocardial infarction; LAD: Left anterior descending coronary artery; HBFP: Hematoxylin-basic fuchsin-picric acid staining.

## Competing interests

The authors declare that they have no competing interests.

## Authors’ contributions

HTB carried out IHC staining and wrote the manuscript. YY carried out the ELISA experiments. JYH participated in IHC staining. YML performed the statistical analysis and participated in evaluation of IHC. CLM was responsible for critical revision of the manuscript and was involved in drafting it. BC conceived the study, participated in its design, and helped draft and edit the manuscript. All authors read and approved the final manuscript.
